# When to Eat

**Published:** 1893-01

**Authors:** James H. Jackson


### WHEN TO EAT.

It is a debatable question how often food is required to preserve health or aid in restoring it, but the important point is gained when sufficient nourishment is supplied to make good the continual waste of tissue, thus keeping the body in repair and maintaining the normal expression of vital forces.

To fulfill the demands of the system, the blood should be kept in condition to nutrify all the tissues of the body, neither falling below nor rising above a healthful standard of nutritive power.

As the process of nutrition, involving the removal of waste and the assimilation of new material, take place more rapidly in some cases than in others, no absolute rule can be laid down as to the frequency of eating, but, in general, it may be said that nourishment must be adequate to prevent deterioration of the blood. The average man in active life requires daily from 16 to 23 ounces of food, free from water, representing three or four pounds of the ordinary nutritive substances. Now, scientifically, it would seem to be of little consequence when this amount is taken into the system, but practically, it does make considerable difference. First, it is necessary to prevent exhaustion of the nutritive elements in the blood, and, second, to guard against any functional digestive disturbance, thus precluding an abnormal manifestation in the organs of digestion. In other words, if the demands upon the digestive functions are too frequent there is a liability to irritation, exhaustion, inflammation, and the whole train of functional disorders. Overloading the stomach tends to produce the same or similar conditions.

Habit largely controls the time of eating, but habit is often formed without regard to any necessity on the part of the organism, and may be arranged with equal facility to longer or shorter intervals, as may be desired.

The quality of food, also, has an important bearing upon the subject. Persons who live on stimulating articles of diet, including tea, coffee, animal flesh in considerable quantities, condiments, or alcoholic stimulants, seem to requre food oftener than those who choose an unstimulating diet. The reason is that regardless of the nutritive material already in the blood, a seeming demand exists for an additional quantity, which is expressed by a sense of hunger or weakness as a result of reaction from the stimulants taken ; and this reaction usually asserts itself more imperatively than the natural desire for food, when there has been no undue stimulation. True hunger is always a call from the tissues of the body for nutritive material, while much of the so-called hunger is merely an expression of irritation of the nerves of the stomach arising from inflammatory or irritative conditions as the result of reaction from previous stimulation.

Persons living on stimulating foods, when called upon to forego any accustomed meal, complain of weakness, "goneness," and hunger to a greater degree than those who live on an unstimulating diet.

As a rule, meals occur too frequently, compelling the organs of digestion to undergo the physiological congestion which follows the introduction of food into the stomach so often that after a longer or shorter period a pathological or abnormal congestion is set up which results in lack of secretion, or changes the character of the fluid contents of the stomach to such an extent that fermentation and the formation of gas follow. Unquestionably the majority of people would be better in health to take but two meals a day, breakfast at eight or nine o'clock in the morning, and dinner at five or six in the afternoon. Years of personal experience in the treatment of invalids, and in the observations of healthy persons who have recovered from functional disturbances by adopting a correct diet and two meals a day, in place of three or five, together with the added corroboration afforded by the experiences of many persons living for years in healthful activity on two meals a day, are convincing arguments in favor of less frequent eating. There are innumerable instances to prove that the majority of persons would gain in health and strength upon two meals a day. But whatever the habit may be as to the number of meals, there is no more pernicious practice than that of eating between meals either by children or adults, and in the course of time it is sure to impair digestion. The lack of knowledge and conscience shown in this regard by the average man or woman is deplorable. It would seem as if the stomach was thought to be made of cast-iron, and therefore incapable of being exhausted or irritated by overwork, and that all kinds of substances could be put into it with impunity at any hour of the day or night. Persons who live after this haphazard fashion are amazed to find after a while that they are suffering from serious digestive disturbances, and the physician's protest against dietetic sins is often met with indifference and disregard.

But it must be borne in mind by those who seek to preserve health, or recover it when lost, that regularity is second in importance to no other factor. It is true that one may sometimes continue for years to violate physiological law, yet because a regular habit has been established the consequences are less serious than they would be had the same infringement occurred at regular intervals. As regularity is so potent an influence in securing health, happiness, and success, it is the part of wisdom to establish a rule as to the number of meals and the times of eating, and then hold with conscientious rectitude to the observance of the rule. If this is done and the happy mean is maintained between over-eating on the one hand, and semi-starvation on the other from an insufficient supply of food or that which is poor in quality, the result will be in favor of sound health and freedom from the bondage of mental and physical distresses which follow in the train of digestive disturbances.—*James H. Jackson, M.D., in Laws of Life.*

